# Co-delivery of deferoxamine and hydroxysafflor yellow A to accelerate diabetic wound healing via enhanced angiogenesis

**DOI:** 10.1080/10717544.2018.1513608

**Published:** 2018-10-19

**Authors:** Si-Qian Gao, Chen Chang, Jun-Jun Li, Ying Li, Xiao-Qian Niu, Dan-Ping Zhang, Long-Jian Li, Jian-Qing Gao

**Affiliations:** a Institute of Pharmaceutics, College of Pharmaceutical Sciences, Zhejiang University, Hangzhou, Zhejiang, P.R. China;; b Zhejiang Provincial Corps Hospital of Chinese People's Armed Police Forces, Jiaxing, Zhejiang, P.R. China;; c Jiangsu Engineering Research Center for New-Type External and Transdermal Preparations, Changzhou, P.R. China

**Keywords:** Hydroxysafflor yellow A, deferoxamine, diabetic wound healing, angiogenesis, hydrogel

## Abstract

Nonhealing chronic wounds on foot induced by diabetes is a complicated pathologic process. They are mainly caused by impaired neovascularization, neuropathy, and excessive inflammation. A strategy, which can accelerate the vessel network formation as well as inhibit inflammatory response at the same time, makes it possible for effective diabetic ulcers treatment. Co-delivery of multiple drugs with complementary bioactivity offers a strategy to properly treat diabetic wound. We previously demonstrated that hydroxysafflor yellow A (HSYA) could accelerate diabetic wound healing through promoting angiogenesis and reducing inflammatory response. In order to further enhance blood vessel formation, a pro-angiogenic molecular called deferoxamine (DFO) was topically co-administrated with HSYA. The *in vitro* results showed that the combination of DFO and HSYA exerted synergistic effect on enhancing angiogenesis by upregulation of hypoxia inducible factor-1 alpha (HIF-1α) expression. The interpenetrating polymer networks hydrogels, characterized by good breathability and water absorption, were designed for co-loading of DFO and HSYA aiming to recruit angiogenesis relative cells and upgrade wound healing *in vivo*. Both DFO and HSYA in hydrogel have achieved sustained release. The *in vivo* studies indicated that HSYA/DFO hydrogel could accelerate diabetic wound healing. With a high expression of Hif-1α which is similar to that of normal tissue. The noninvasive US/PA imaging revealed that the wound could be recovered completely with abundant blood perfusion in dermis after given HSYA/DFO hydrogel for 28 days. In conclusion, combination of pro-angiogenic small molecule DFO and HSYA in hydrogel provides a promising strategy to productively promote diabetic wound healing as well as better the repair quality.

## Graphical Abstract

**Figure:**
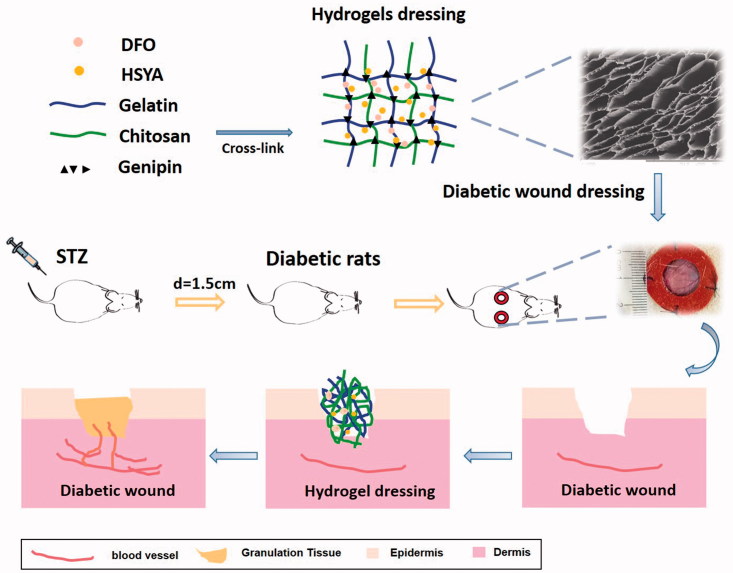

## 1. Introduction

By 2030, approximately more than 489 million adults worldwide would be afflicted with diabetes (Blakytny and Jude, 2006). There is a high mortality for diabetic patients who suffer from chronic ulcers, because these nonhealing wound on foot is the major cause of non-traumatic amputations, which carry a 50% death rate on average due to infection (Barshes et al., 2013; Wukich et al., 2013). Although many therapeutics have been proposed to improve diabetic ulcers, only a few modestly effective technologies are now clinically applied.

Diabetic ulcers have a complex pathologic process, within which a major factor leading to nonhealing wound in diabetes is the reduced neovascularization caused by hyperglycemia (Faglia et al., 2013). Rich blood flow helps transfer nutrients to the wound site and insufficient local blood perfusion at the distal part of body leads to limb ischemia and even tissue necrosis (Capla et al., 2007; Jan et al. 2013). Therefore, accelerating the vessel network formation is of importance. Nowadays, there are many vascular remodeling based researches (Cho et al., 2006; Hou et al., 2013; Kant et al., 2015; Gao et al., 2017; Wang et al., 2018). We discovered a Chinese medicine monomer could effectively accelerate wound healing mainly depends on vascular promotion (Chen et al., 2012; Shan et al., 2015; Li et al., 2017), which makes it a potential drug for trauma treatment. We also demonstrated that hydroxysafflor yellow A (HSYA), a chief bioactive compound derived from the herb *Carthamus tinctorius* L., could speed up diabetic wound healing through reducing inflammatory response, enhancing angiogenesis and re-epithelialization (Gao et al., 2018). But its effect on bettering the repair quality is still limited, since HSYA alone is not enough to exert angiogenesis effect on the diabetic wound healing. We tried to increase the dosage of HSYA to enhance its effect on diabetic wound healing, but the result was counterproductive (Supplementary Figure S1), which is probably because high concentration of HSYA inhibits angiogenesis (Wang et al., 2016). Hence, we consider synthetically combining two drugs to promote diabetic wound healing more efficiently through enhancing angiogenesis.

It has been reported that deferoxamine (DFO), a FDA-approved pro-angiogenesis small molecule, could correct the impaired HIF-1αmediated transactivation in diabetes (Andrews, 1999). DFO can chelate iron by binding ferrous (Fe^2+^) with the hydroxy groups to prevent iron-catalyzed reactive oxygen stress (Sarkar et al., 2009), so as to prevent the degradation of HIF-1α. HIF-1α is critical in the regulation of cellular oxygen homeostasis and responses to hypoxia. In normal wound healing process, it is up-regulated to promote the expression of major angiogenesis relative cytokines, such as vascular endothelial growth factor (VEGF) and stromal cell-derived factor 1 (SDF-1α) (Ceradini et al., 2004; Thangarajah et al., 2009; Thangarajah et al., 2010). However, the compromised function of HIF-1α in diabetes, which is induced by high glucose and reactive oxygen species (ROS), leads to impaired HIF-1α transactivation and reduced neovascularization (Duscher et al., 2015; Rabbani et al., 2017). DFO could promote diabetic ulcers repair through up-regulating the expression of HIF-1α. Therefore, we combine HSYA with DFO as a possible efficient strategy for chronic ulcers treatment to further strengthen the effects of HSYA on angiogenesis.

Since problems such as drug lost or wound infection may happen when drug solution is directly applied to the skin, we need to find a proper carrier to incorporate the drug monomer. Hydrogel is one of the most widely used dressings for diabetic wounds in clinical (Sun et al., 2011; Amin and Abdel-Raheem, 2014; Tran et al., 2011). Compared with the traditional dressings, hydrogels have interconnected pores which facilitate drug release and wound exudates absorption. Moist environment caused by hydrogels lead to accelerated granulation and re-epithelialization (Okan et al., 2007). Chitosan is a nontoxic cationic polysaccharide derived from partial N-deacetylation of chitin, possessing excellent biodegradability, antibacterial, and hemostatic properties. These characteristics make it a potentially candidate biomaterials for tissue regeneration and wound healing (Bhattarai et al., 2010). However, chitosan hydrogels alone are limited by its unamiable surface and mechanical weakness (Park et al., 2010). To address these problems, another natural polymer - gelatin - were employed to cross-link with chitosan and also Genipin, a low toxic natural cross-linker, was used for hydrogel formation (Cui et al., 2015). Herein, we incorporated HSYA and DFO into the interpenetrating polymer networks (IPN) hydrogels fabricated by gelatin and chitosan to investigate whether it can accelerate diabetic wound healing.

## 2. Materials and methods

### 2.1. Materials

HSYA and Genipin (GP) were purchased from Shanghai Sunny Biotech Co., Ltd. (China; purity >98%, high-performance liquid chromatography (HPLC)). Deferoxamine (DFO) was obtained from Sigma-Aldrich (St.Louis, MO). Chitosan (viscosity: 100–200mPas; degree of deacetylation: >95%), gelatin type B (from bovine skin), paraformaldehyde, Triton X-100 and thiazoylblue tetrazoliumbromide (MTT) were obtained from the Sigma-Aldrich.Cell Counting Kit-8 (CCK-8)assay kit and nitric oxide (NO) assay kit were purchased from Boster, Inc. (Wuhan, China). Matrigel was purchased from BD Biosciences (Franklin Lakes, NJ). The immortalized human keratinocytes line, human fibroblast cells, and human umbilical vein endothelial cells were obtained from the institute of Biochemistry and Cell Biology, Shanghai Institution for Biological Sciences, Chinese Academy of Science.

### 2.2. Animals

Six-week-old male Sprague-Dawley rats (220–240g) were applied in diabetic wound healing studies. All animals were supplied by and kept in Zhejiang University Experiment Animal Center, China. They were maintained under the condition of 25 ± 1 °C with free access to standard animal food and tap water. All animal experiments were carried out according to the Zhejiang University guidelines concerning welfare of experimental animals.

### 2.3. *In vitro* studies

#### 2.3.1. Wound scratch assay

The Culture Insert (ibidi GmbH, Martinsried, Germany) was used to create a wound in the cell culture (Zhu et al., 2015). The culture insert was placed on a 24-well plate and 70 µL of human epithelial keratinocytes (HEKs) at a concentration of 5 × 10^5^ cells/mL was added to both wells of the insert. After 24 h, the culture was wounded by removing the existing insert which leave a scratch and medium supplemented with HSYA solution and HSYA/DFO solution were added to continue the cell culture and wound healing process. All scratches were performed in triplicate. The migration of keratinocytes was photographed using an inverted microscope at 0, 60, and 96 h until gap closure was complete. The percentages of wound closure were calculated using the ImageJ software (NIH, Bethesda, MA). An increase in the percentage of the closed area demonstrated the migration of cells.

#### 2.3.2. Tube formation assay

The Matrigel basement membrane matrix was thawed at 4 °C overnight. All pipettes and 96-well culture plates were pre-cooled before use. After coating the plates with Matrigel (50 µl/well) for 30 min at 37 °C until it solidified, medium supplemented with various concentrations of HSYA or DFO containing 1.5 × 10^4^ human umbilical vein endothelial cells (HUVECs) were seeded in each well. After 4–6 h, tube-like structures formed and calcein was continually added to the culture for 30 min. Then fluorescence was photographed by an inverted fluorescence microscope. The total tube length was quantified using the ImageJ software.

#### 2.3.3. Cellular immunofluorescence assay

Human dermal fibroblasts were seeded in 24-well plates. After culturing in 37 °C and 5% CO_2_ humidified incubator for 24 h, the cells were washed three times with PBS (pH 7.4) and then fixed with 4% paraformaldehyde for 10 min at room temperature. After permeabilized with 0.2% (v/v) Triton X-100 for 15 min, the cells were blocked with 1% bovine serum albumin (BSA) for 30 min and incubated with primary anti-HIF-1α (1:50) at 4 °C overnight. After rinsing with PBS for three times, the cells were incubated with Fluorescein isothiocyanate (FITC) -labeled anti-mouse IgG (1:500) for 1 h at room temperature in darkness and then counterstained with 4′,6-diamidino-2-phenylindole (DAPI) for 15 min.

#### 2.3.4. Preparation of HSYA/DFO chitosan/gelatin hydrogels

Firstly, chitosan was dissolved in deionized water containing 1% (v/v) acetic acid at room temperature to obtain a 3% (w/v) solution. Gelatin was dissolved in deionized water at 50 °C to obtain a 2.5% (w/v) solution. Then, the chitosan and gelatin were mixed in different ratio (3:7,5:5,7:3) and stirred for 4 h at 50 °C. The hydrogels were prepared by blending chitosan and gelatin solution with genipin (0.5%), followed by stirring for 6 h at 40 °C. HSYA and DFO were added to the hydrogel with stirring at 40 °C for homogenization (the final concentration is 2 and 0.4 mg/mL, respectively). Blank hydrogel was also prepared as the above procedure.

#### 2.3.5. Scanning electron microscope (SEM) observation

Lyophilized hydrogels were carefully sectioned, fixed on a metal holder and coated with gold. The cross-section morphology of the hydrogel was observed by using a scanning electron microscope (JSM-5510LV, JEOL, Japan).

#### 2.3.6. Rheology test

An R/S plus Mo8-219 rheometer (Brookfield, MA) was used to quantitatively evaluate the rheological behavior of chitosan/gelatin hydrogel by dynamically monitoring variations of hydrogel storage modulus (G′) and loss modulus (G″) as a function of frequency (Hz). HSYA-DFO loaded hydrogel was spread onto a parallel plate (diameter: 40 mm) for testing in accordance with standard practice D and the concerned parameters were evaluated at 37 °C.

#### 2.3.7. *In vitro* drug release profile

The drug release study was performed using the dialysis bag method. Briefly, a dialysis bag (molecules cutoff of 8000–14,000) was required to be soaked in the release medium overnight prior to the research. The HSYA solution and HSYA-gel, placed in dialysis bags, were immersed in centrifugal tubes containing 8 ml PBS (pH7.4) at 32 °C at 100 rpm for 60 h to assure the maximum drug release. At fixed intervals, the release medium was withdrawn from the centrifugal tubes and replaced with fresh release medium to maintain the sink condition. The cumulative amount of HSYA released was determined by HPLC.

### 2.4. *In vivo* diabetic wound healing study

#### 2.4.1. Establishment of a diabetic rat model

Six-week-old Sprague-Dawley rats (220–240g) were used for the study. After adaptive feed for two weeks, 270–300 g rats were induced for Type 1 Diabetes Mellitus (T1DM) model by a single intraperitoneal injecting streptozotocin (STZ) at 75 mg/kg. STZ was dissolved in citrate buffer (pH 4.2–4.5). Three days later, the tail vein blood glucose levels were measured. Those rats with a blood glucose level over 16.7 mmol/L were considered as diabetic rats. Their blood glucose and body weight were detected for five weeks before surgery.

#### 2.4.2. Establishment of a diabetic wound model

Diabetic rats were randomly chosen and divided into four groups: Control (PBS), HSYA/DFO solution, blank hydrogel and HSYA/DFO hydrogel (six wounds per group). The animals were anesthetized and the hair of their backs was shaved with an electric clipper. Their skin was wiped with 70% alcohol. Then, two 1.5 cm, circular, full-thickness excisional wounds that extended through the panniculus carnosus were made on each side of the midline with a biopsy punch. Next, 1-mm-thick silicone donut-shaped splints were fixed around the wounds and positioned with 6-0 nylon sutures to prevent contraction (Chereddy et al., 2015; Wang et al., 2013).

#### 2.4.3. *In vivo* diabetic wound repair experiment

HSYA (2 mg/mL), DFO (0.4 mg/mL), HSYA/DFO solution, and PBS solution (2/0.4 mg/mL) were applied at the wound site once a day. Wounds on the same mouse received different types of treatments. The rats were individually housed and digitally photographed every three days. The wound areas were quantified using Image-J software and calculated as a percentage of the initial wound areas. Then we conducted the experiment about HSYA/DFO hydrogel. There were a total of four groups: 2/0.4 mg/mL of HSYA/DFO solution, 2/0.4 mg/mL of HSYA/DFO hydrogel (the ratio of GL/CS is 5:5), PBS solution, and a blank hydrogel. HSYA/DFO hydrogel was applied once every two days.

#### 2.4.4. Evaluation of the regenerated tissue via histology and immunofluorescence

The skin tissues harvested on day 28 were fixed in 4% buffered paraformaldehyde overnight and embedded in paraffin, then sectioned (5um) and processed for hematoxylin and eosin (HE) staining to measure granulation tissue thickness, epidermis thickness. The tissue sections were also processed for immunofluorescence staining of cluster of differentiation 31 (CD31) and HIF-1α to investigate the content of newly formed vascular. Inverted fluorescence microscope was used for photograph and Image J software was applied for quantitative analysis of the samples.

#### 2.4.5. Blood flow measurements at the wound sites

The signals of ultrasound (US) and photoacoustic (PA) imaging signals were collected using an ultrasound imaging system (Vevo 2100; Visual Sonics, Inc., Toronto, Canada) with ultrasound array transducer (MS-250 or MS-550S; Visual Sonics, Inc.). Tunable pulsed laser systems (Premiscan; GWU, Inc.( Santa Clara, CA) and Vevo LAZR; Visual Sonics, Inc.) were used to deliver photons through an optical fiber bundle. The blood perfusion from diabetic wounds at the day 28 were evaluated. While measuring, the diabetic rats were anesthetized and placed on a heating plate at 37 °C to maintain the body temperature.

### 2.5. Statistical analysis

Numerical data are reported as mean ± standard deviation (SD) of the mean. The statistical significance between two sets of data was calculated using a two-tail Student's *t*-test. One and two way analysis of variance (ANOVA) tests were used to measure differences for experiments with multiple datasets with a Turkey test performed between groups with significant differences to correct for the multiple pair-wise comparisons. A probability (*p*) value <.05 was considered significant.

## 3. Results and discussion

### 3.1. Co-culture of HSYA and DFO promoted cell migration and tube formation

**Figure 1.**
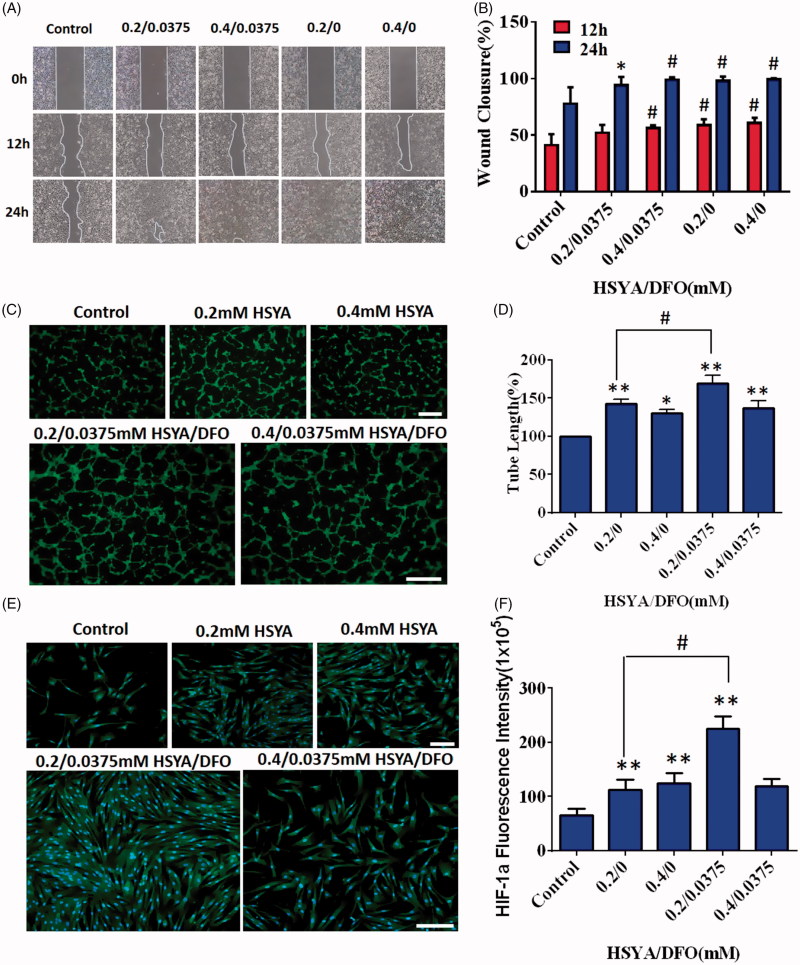
(A) Effects of DFO/HSYA on migration of HaCats were assessed by an IBIDI wound healing assay from 0 to 24h. (B) Wound closure of HaCaTs were expressed as a percentage of the initial wound. *p<.05, #*p*<.01 versus control (*n* = 9; mean ± SD). (C) Fluorescent images of tube formation assay in three-dimensional Matrigel after treatment of HSYA/DFO for 6h (scale bars: 500um, magnification: 10x). (D) Quantitative analysis of the total tube length in HSYA-treated HUVECs using the ImageJ software. (E) Cellular immunofluorescence staining of HIF-1α in human dermal fibroblasts (scale bars: 200um). (F) Quantification of the cellular immunofluorescence. *p<.05 and **p<.01 versus control; #*p*<.01 versus HSYA alone (*n* = 3; mean ± SD).

As shown in Figure 1, HSYA alone could dose-dependently promote cell migration (*p* < .05). When HSYA was co-applicated with DFO, significant migration was also observed. After 24 h incubation, HEKs demonstrated nearly complete scratch gap closure (94.1 and 99%) compared with control (77.8%) (Figure 1(B)). Therefore, the presence of DFO did not affect the bioactivity of HSYA. Migration of keratinocytes is a critical step for re-epithelialization (Moura et al., 2013). Improved migration could accelerate the wound healing process.

Angiogenesis is a pivotal process for wound repair, which facilitates the delivery of oxygen, nutrients, and related growth factors to wound bed, promoting formation of granulation tissues (Martin et al., 2003). It was observed that HUVECs with addition of 0.0375/0.2 mM of HSYA/DFO formed more branched tubular structure at 6 h when compared with control (Figure 1(C)). As observed in Figure 1(C), HSYA alone (0.4 mM) significantly boosted the capillary-like structures formation (*p* < .05). Although lower concentration (0.2 mM) of HSYA resulted in more tube formation, the combination of low concentration of HSYA and DFO presented much stronger enhancement on tube formation than HSYA treatment alone, whose average length of complete tubes was the highest (Figure 1(D)).

HIF-1α was expressed in both the cytoplasm and nucleus according to the cellular immunofluorescence images, but the majority of HIF-1α was distributed in the nucleus (Figure 1(E)). As shown in Figure 1(F), after 24 h of culturing with HSYA/DFO, significant up-regulation of HIF-1α was observed in cells compared with control (*p* < .05). There was higher HIF-1α expression in 0.0375/0.2 mM HSYA/DFO groups compared with the 0.2 mM HSYA alone. *In vitro* experiments, we found that HSYA/DFO significantly increased tube formation and secretion of HIF-1α when compared with HSYA alone. The underlying mechanism of diabetes-induced impairments in neovascularization is a dysfunction of HIF-1α. DFO, as an iron chelator, can purely upregulate HIF-1α levels in dermal fibroblasts. High expression of HIF-1α in fibroblasts recruit more angiogenesis relative cells and secrete major angiogenesis relative cytokines (VEGF, SDF-1α) (Liu et al., 2014). VEGF and SDF-1α are essential for vascular development, which increases the proliferation and migration of endothelial cells, recruits circulating endothelial progenitor cells and improves angiogenesis, and thus leading to more vessels formation (Bergeron et al., 2003), which is beneficial for diabetic wound healing. DFO has also been reported as a direct antioxidant and can reduce the oxidative stress associated with ischemia (Sundin et al., 2000). Therefore, DFO plays a protective role during hypoxic preconditioning in brain (Prass et al., 2002) and heart tissue (Dendorfer et al., 2005) as well as in cutaneous ischemic preconditioning. In addition, it has been reported that HSYA enhance endothelial cell survival under hypoxia by up-regulating the HIF-1α-VEGF pathway (Ji et al., 2009). It means that HSYA could significantly increase the expression of VEGF through up-regulation of HIF-1α. It is consistent with that of DFO.

### 3.4. Preparation and characterization of chitosan/gelatin hydrogel

Exposure of diabetes ulcers to the air can rise the infection risk. Appropriate dressings are needed to prevent diabetic wound from bacterial infections and absorb wound exudates. Several technologies such as silicone-coated foam and hydrocolloids have been used to reduce the risk of ulcer formation (Dumville et al., 2013). A diabetic nonhealing wound requires repetitive treatment administration and DFO has a short biological half-life (Hom et al., 2000). However, frequent dressings may result in increased pain and irritability. To reduce application frequency and increase safety as well as maintain drug efficacy, it is ideal to design a formulation with sustained drug release. Hydrogels are widely used in the clinical treatment for diabetic wounds. They have interconnected pores which facilitate drug release and wound exudates absorption. Hydrogel with excellent biocompatibility provides extracellular matrix for cells to function well. Thus, hydrogel is considered as a suitable carrier for topical application. Herein, we made the interpenetrating polymer networks hydrogels based on gelatin and chitosan to reduce application frequency and thereby increase patient compliance and convenience.

**Figure 2.**
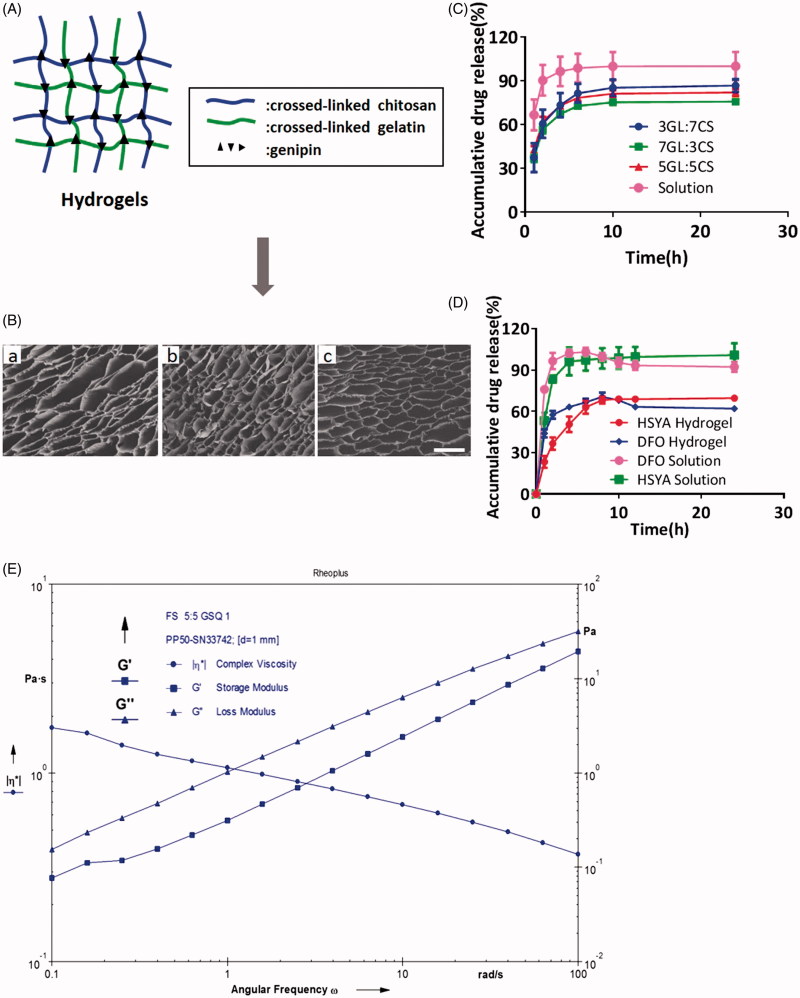
(A) Schematic diagram of IPN porous structure using chitosan and gelatin. (B) SEM images of HSYA hydrogels produced at a variety of GL/Chi ratio; Scale bars: 200um; (a) 3:7, (b) 5:5, (c) 7:3, and (d) 7:3. (120×). (C) Cumulative release profiles of HSYA-loaded hydrogels. (*n* = 3; mean ± SD). (D) Cumulative release profiles of HSYA-DFO hydrogels. (GL/Chi ratio = 5:5; *n* = 3; mean ± SD). (E) Rheology test of HSYA-DFO hydrogels. (G’: storage modulus, G”: loss modulus).

As shown in Figure 2(A), chitosan and gelatin molecule chains permeate each other fixing by crosslinker GP, resulting in the formation of sponge-like interpenetrating polymeric networks (IPN). The crosslinked hydrogels exhibited a well-defined, three-dimensional porous structure with interconnected pores (Figure 2(B)). These pores had orderly and thin wall, which provided surface for cells like HSFs and HEKs to attach to or proliferate. It was observed that diverse ratio of GL/CS had little influence on the size of interconnected pores (Figure 2(B)). Hydrogels that are constitute of different ratio showed no significant released profile (Figure 2(C)).

After incorporating HSYA and DFO into hydrogel, we found that the release rate of DFO was always faster than that of HSYA. It is probably because DFO has smaller molecular weight (Figure 2(D)). In Figure 2(D), Both DFO and HSYA in solution showed a burst release before 2 h which was about 97 and 84% respectively. When they were loaded in hydrogel, they achieved sustained release. DFO in hydrogels kept the initial period of fast release after 1 h (about 60–70%), then followed by sustained release in later point. And the platform period of hydrogel was achieved by 8 h while the solution happened at 4 h. HSYA in hydrogel kept the initial period of fast release after 1 h (about 40–50%) whereas followed by sustained release until 10 h. Both HSYA and DFO in hydrogel achieved fast release at early stage, which allow the diabetic wound to be covered with plenty of drugs so that the drug can better chronic wound healing at early stage.

Rheology test has been broadly employed in the evaluation of hydrogel viscoelastic properties, with storage modulus G′ representing the elasticity part and loss modulus G″referring to the viscosity part of hydrogel. Figure 2(E) shows the changes in storage modulus and loss modulus of HSYA/DFO hydrogel as a function frequency. It showed that HSYA/DFO hydrogel was pseudoplastic fluid and it had yielding behavior, which confirmed that drug loaded hydrogel stayed at gel phase at 37 °C. Thus, it is suitable dressing that can be topically applied on the skin for diabetic ulcers treatment.

### 3.5. Co-loaded hydrogel accelerated wound closure in STZ-induced diabetic rats

**Figure 3.**
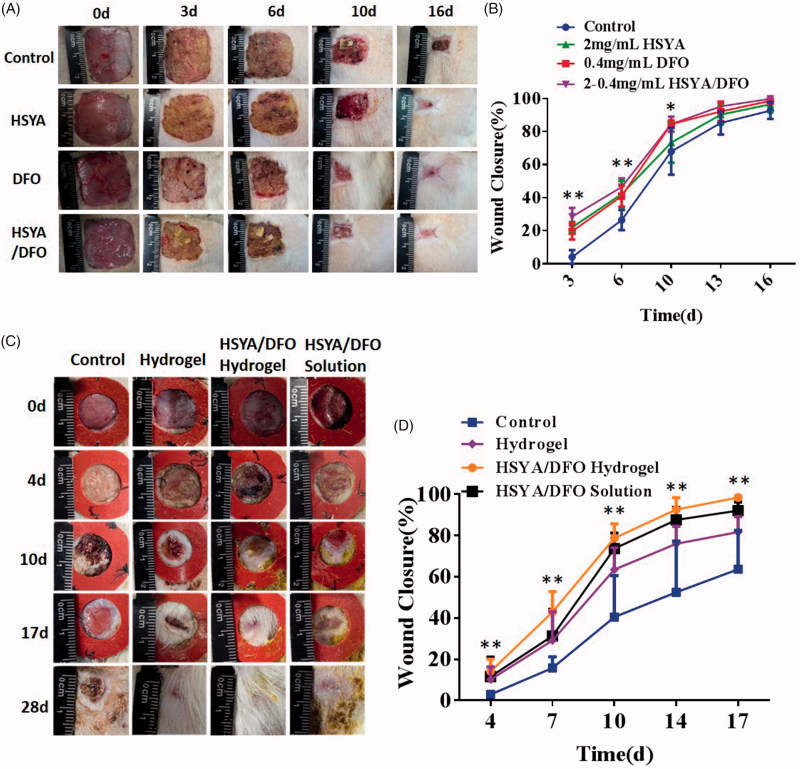
(A) Representative images of wound healing a particular time point. (B) Quantitative wound closure percentage at a particular time point using the ImageJ software. (C) Representative images of wound healing a particular time point. (D) Quantitative wound closure percentage at a particular time point using the ImageJ software. *p < .05 and **p < .01 versus control (*n* = 6; mean ± SD).

DFO is an iron chelating agent that increases HIF-1α transactivation in diabetes by eliminating iron-catalyzed ROS. It can promote wound healing and decrease tissue necrosis in the setting of diabetes. Based on the pro-angiogenesis activity of HSYA/DFO co-administration on HSFs and HUVECs, we investigated whether HSYA/DFO could enhance diabetic wound healing in rat models. In our *in vivo* experiment, a splinted rat full-thickness excisional model was used to evaluate the diabetic wound healing effects. As we know, contraction leads to rapid closure of rodent wounds, and it has been reported that unsplinted wounds in db/db mice recover two times faster than the splinted (Michaels et al., 2007). Splinting prevents rapid contracture of the wounds and allows differences in granulation tissue formation or epithelialization to be assessed accurately over time (Wang et al., 2013). Therefore, our studies used this model. As shown in Figure 3(A), the more significant enhancement effect of HSYA/DFO in chronic wound repair was observed compared with the control and HSYA alone group. It demonstrated that combined application of HSYA and DFO had a synergistic effect and that could accelerate diabetic wound healing. At the beginning, DFO was widely used in patients with Mediterranean anemia to chelate redundant Fe^2+^ in blood and spleen. Until recently, attention has been paid to its pro-angiogenesis functions in bone and soft tissue (Ihnat et al., 2000). However, the application of DFO in treating wound defects have been rarely reported. It was the first time that we found co-administration of HSYA and DFO was effective in promoting wound healing in diabetic rats.

As shown in Figure 3, at day 4, there is no significant difference in wound closure among treatment group, because the new vessels were not formed and major of relative dermal cells have not migrated into the wound site in the early condition (Sharma et al., 2018). However, at day 7, the wound closure of HSYA/DFO hydrogel reached highest, which was nearly 42.9% while PBS was 14.7%. At early stage, fast drug release is beneficial for wound healing. With fast release of HSYA and DFO from the hydrogel at early stage, plenty of drugs covered wounds to function well. In addition, early scarring and epithelialization provided barriers to prevent infections. The wounds treated with HSYA/DFO hydrogel were dry and easier to cause scar formation during the healing process. From the day 10 to 14, the healing rate of treatment group increased rapidly as a result of the falling off of the hard scar. The PBS-treated wounds healed completely at almost the day 32 when that happened at day 17 with HSYA/DFO hydrogel. The healing rate of HSYA/DFO solution was not as good as HSYA/DFO hydrogel. HSYA/DFO solution is easily to loss. Although there were sufficient drugs on wounds at early stage, they could be degraded in the skin quickly without new drug supplement, they have short plasma half-life (Hom et al., 2000; Jin et al., 2016). In contrast, HSYA and DFO in hydrogel could take effect continually during wound healing process due to sustained release. Although the sustained release of hydrogel is not obvious, there is less exudate at the wound site, the actual release of the drug from hydrogel is slower. In addition, the maximum drug release didn’t appear around 10 h, it was just a platform period, after which the drug could still release continually although the speed is slow. Therefore, the drug release time could be far more than 10 h. In order to maintain the effect of HSYA/DFO, we used HSYA/DFO hydrogel every two days according to the drug release while HSYA/DFO solution was used every day. In this case, the results showed the advantage of combining HSYA with DFO in hydrogel on better accelerating diabetic wound repair. The healing rate reached nearly 100% in two weeks with fewer usage frequency. As high expression of HIF-1α in fibroblasts induced by combined application of HSYA and DFO continually, more angiogenesis relative cells concentrated and more vessels formed in the wound, significantly accelerating repair process. However, due to the complicated wound healing process, we can’t ensure whether this drug release is optimal, maybe slower release is better, the further study needs to be explored in the future.

### 3.6. Histology and immunofluorescence staining analysis

**Figure 4.**
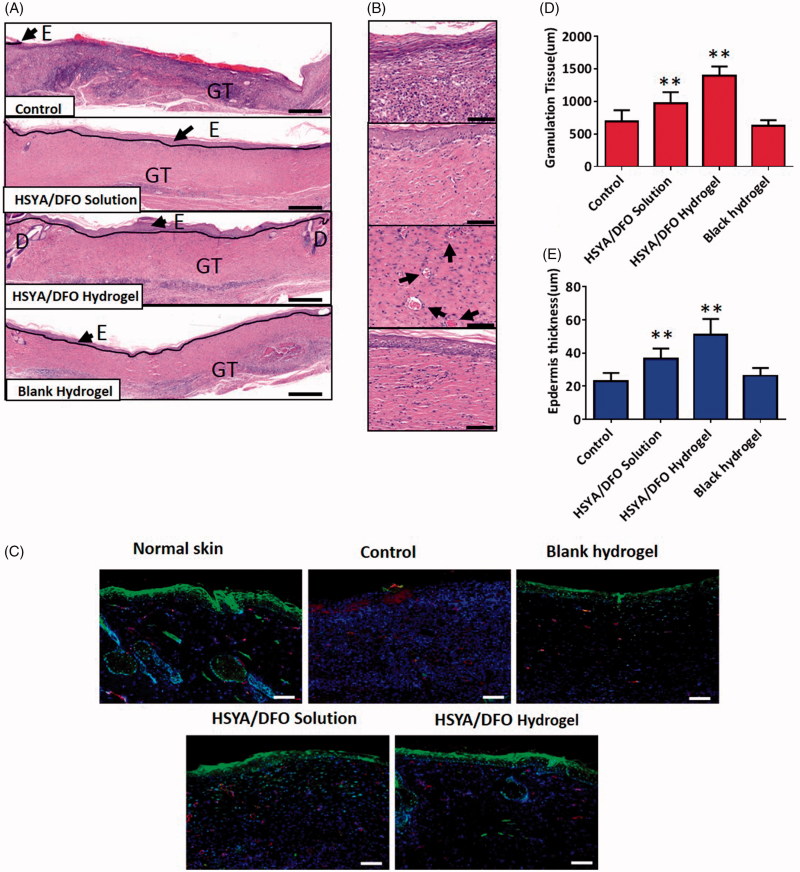
(A) HE stained wound sections at day 28. Scale bars: 200 um. E: epidermis; GT: granulation tissue; D: dermal. (B) High power field (HPF) (40×) imaging of the center of wound granulation tissues. Scale bars, 50 um. Black arrows highlight the newly formed vascular. (C) Immuno fluorescence images for HIF-1α and CD31. Scale bars, 200um. Green: HIF-1α; Red: CD31; Blue: DAPI. (D) Granulation tissue thickness in the center of wounds. (E) Epidermis thickness in the center of wounds. *p < .05 and **p < .01 versus control (*n* = 6; mean ± SD).

Cross-sectional analysis of histological wound sections was used to measure the thickness of new epidermis and granulation tissue at each time point. On day 28, images of histology sections taken from the center part of the wounds showed that wounds treated with PBS has not closed completely yet. So it was not covered by a layer of epidermis completely and exhibited a significantly thinner stratus lucidum and basal layer. When compared to PBS-treated wounds, the HSYA/DFO hydrogel treated wounds were covered with a uniform layer of epidermis (Figure 4(A)). It significantly improved the process of re-epithelialization, leaving proper space for new tissue growth and increased number of capillaries were observed in the dermal layer (Figure 4(A,B)). In addition, newly formed granulation tissue and epidermis were thicker and better structured than other groups, suggesting more rapid healing of the wounds when treated with HSYA/DFO hydrogel (Figure 4(D,E)).

The phenomenon that a strong HIF-1α signal in the epidermal layer of HSYA/DFO treated wounds was observed, indicating a better regenerated and more matured spinous layer. High expression of HIF-1α, which could boost the delivery of nutrients and relative cytokines in fibroblasts, induced more vessels formed in wound bed and improved diabetic wound healing (Figure 4(C)). CD31 was the specific maker of newly formed vascular. On day 28, there is a more similar vessel density and skin structure to normal tissue when treated with HSYA/DFO hydrogel. Overall, proper wound healing requires the formation of a structural and functional epithelial layer and it is mediated by keratinocytes and fibroblasts. Functional neovascularization caused by upregulation of HIF-1α could facilitate cell and nutrition transportation as well as oxygen exchange in wound bed to accelerate wound re-epithelialization. Then the wound treated HSYA/DFO hydrogel resulted in skin regeneration similar with normal skin tissue.

### 3.7. *In vivo* combined US/PA imaging to assess blood vessel formation

Diminished peripheral blood flow and decreased local neovascularization are major factors that initiate the diabetic wounds. Once wounded, impaired angiogenesis further delays the formation of granulation tissue and slows the regeneration process, leaving the wounded area vulnerable to secondary infection and additional injury (Brownlee, 2001). In this study, the microvascular network in the dermis at the wound site was visualized noninvasively via a novel technique. Combined US/PA imaging was used to monitor the blood leakage, caused by blood vessel damage, deep into the skin to the subcutaneous fat (Nam et al., 2015). It has emerged as a tool to rapidly give accurate information about wound depth and blood perfusion. Because hemoglobin can be directly visualized in PA imaging (Tan et al., 2017). There have been numerous studies to visualize and quantify blood vessels using PA imaging for various applications. For example, noninvasive assessment of tumor vascular development using PA imaging has been demonstrated (Plumb et al., 2018) (Yang et al., 2017; 53).

**Figure 5.**
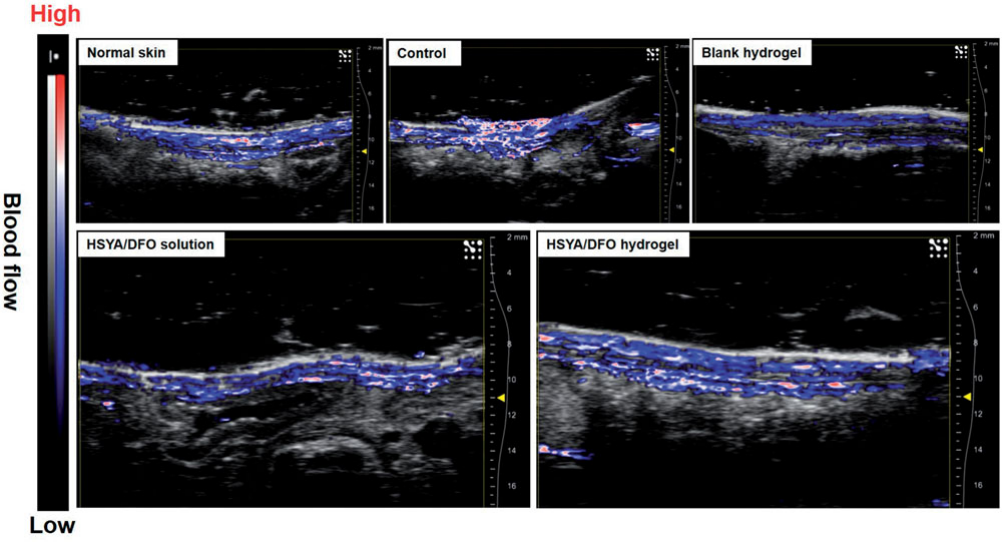
Cross-sectional combined ultrasound and photoacoustic images of diabetic wound for blood flow detection at day 28.

Figure 5 represents the cross-sectional US and PA images at day 28 after the treatment, it can reflect the blood flow at wound site with depth-resolved structural information. In uninjured diabetic rat, there was no bleeding in the subcutaneous tissue while we could observe bleeding throughout the fat tissue. It is the normal blood flow condition of skin. As shown in Figure 5, after being treated with HSYA/DFO hydrogel for 28 days, the wound recovered completely with abundant blood perfusion in dermis. But for other groups like control, the wound was not close completely yet. There was still fluid exudation from damaged tissues and blood vessels, so blood perfusion in the injured region significantly increased. It implies the process of neovascularization involved in the tissue regeneration was not finished. In addition, HSYA/DFO solution and blank hydrogel treated group at day 28 showed fewer blood flow in dermis and their blood perfusion was mainly focus on epidermis. Although there was no significant difference in wound healing rate between HSYA/DFO hydrogel and HSYA/DFO solution group, the quality of wound repair was better when treated with HSYA/DFO hydrogel.

## 4. Conclusions

Re-establishment of the vessel network is very important in the early stage of wound healing in diabetic diseases. In this article, instead of treatment with single drug, we developed the IPN hydrogels for co-loading of HSYA and DFO. We aimed to make use of pro-angiogenesis activity of DFO to enhance the effect of HSYA on wound healing. Specifically, co-administration of DFO and HSYA could promote angiogenesis and up-regulate HIF-1α secretion when compared with using single drug. Moreover, HSYA/DFO hydrogel treated rat exhibited a significantly enhanced tissue regeneration that is more similar to the normal skin. This study for the first time demonstrated co-administration of HSYA and DFO was more effective in promoting wound healing in diabetic rats. Because HSYA and DFO are FDA- approved, this co-loaded hydrogel delivery system is promising for treating diabetic ulcers in clinical.

## Supplementary Material

Supplemental Figure S1
