# Supplementary figures and images for: Co-delivery of deferoxamine and hydroxysafflor yellow A to accelerate diabetic wound healing via enhanced angiogenesis
Source: Drug Deliv. 2018 Oct 19;25(1):1779–89. doi: 10.1080/10717544.2018.1513608 (PMC6201774; doi:10.1080/10717544.2018.1513608)

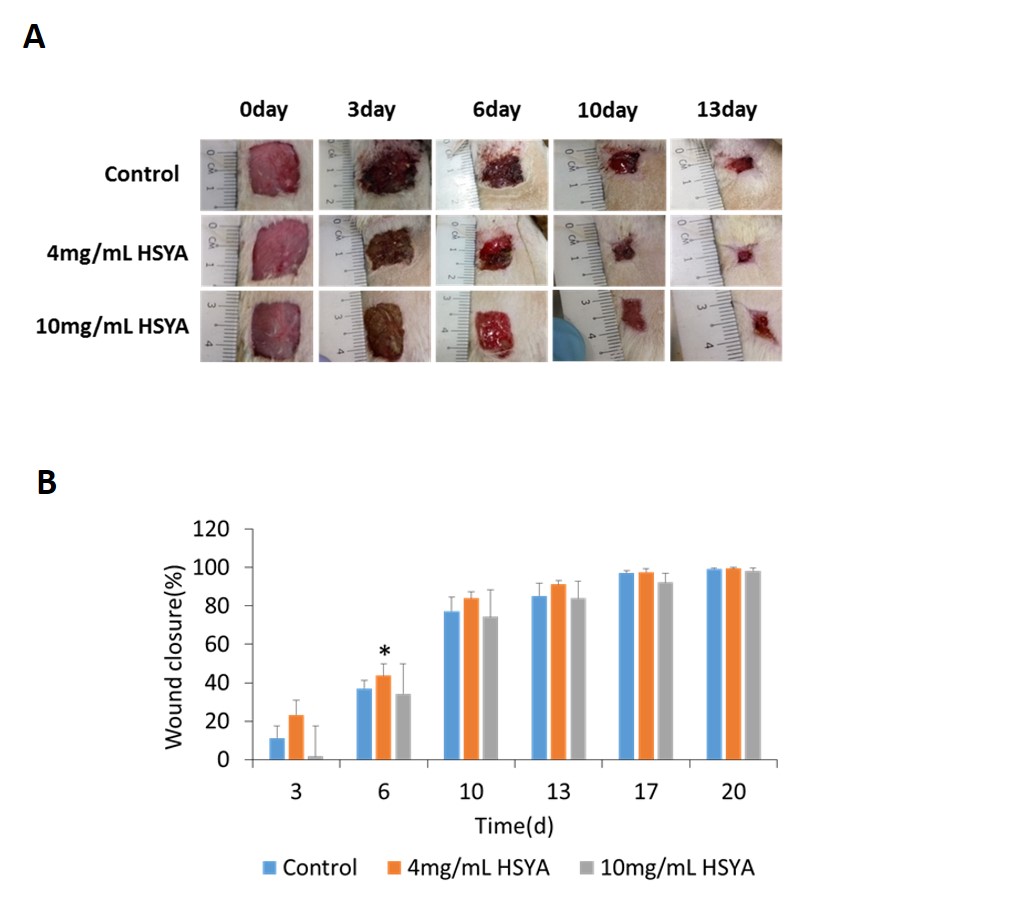

Supplement: Supplemental Figure S1 [file IDRD_A_1513608_SM0256.jpg]
